# Supplementary material for: Engineering metal-carbide hydrogen traps in steels
Source: Nat Commun. 2024 Jan 25;15:724. doi: 10.1038/s41467-024-45017-4 (PMC10808193; doi:10.1038/s41467-024-45017-4)
Supplement: Supplementary file 1 — Supplementary information [file 41467_2024_45017_MOESM1_ESM.pdf]

Supplementary Information for

Engineering metal-carbide hydrogen traps in steels

Pang-Yu Liu[1,2,+], Boning Zhang[3,4,+], Ranming Niu[1,2,+], Shao-Lun Lu[1,5], Chao Huang[1,2], Maoqiu Wang[6], Fuyang Tian[7], Yong Mao[4], Tong Li[8], Patrick A. Burr[9], Hongzhou Lu[10], Aimin Guo[10], Hung-Wei Yen[5,11,*], Julie M. Cairney[1,2,*], Hao Chen[3,*], Yi-Sheng Chen[1,2,5,*]

[+] The authors contribute equally to this work.

[*] Corresponding authors: Yi-Sheng Chen (yi-sheng.chen@sydney.edu.au); Hao Chen (hao.chen@mail.tsinghua.edu.cn); Julie Cairney (julie.cairney@sydney.edu.au); Hung-Wei Yen (homer.yen@ntu.edu.tw)

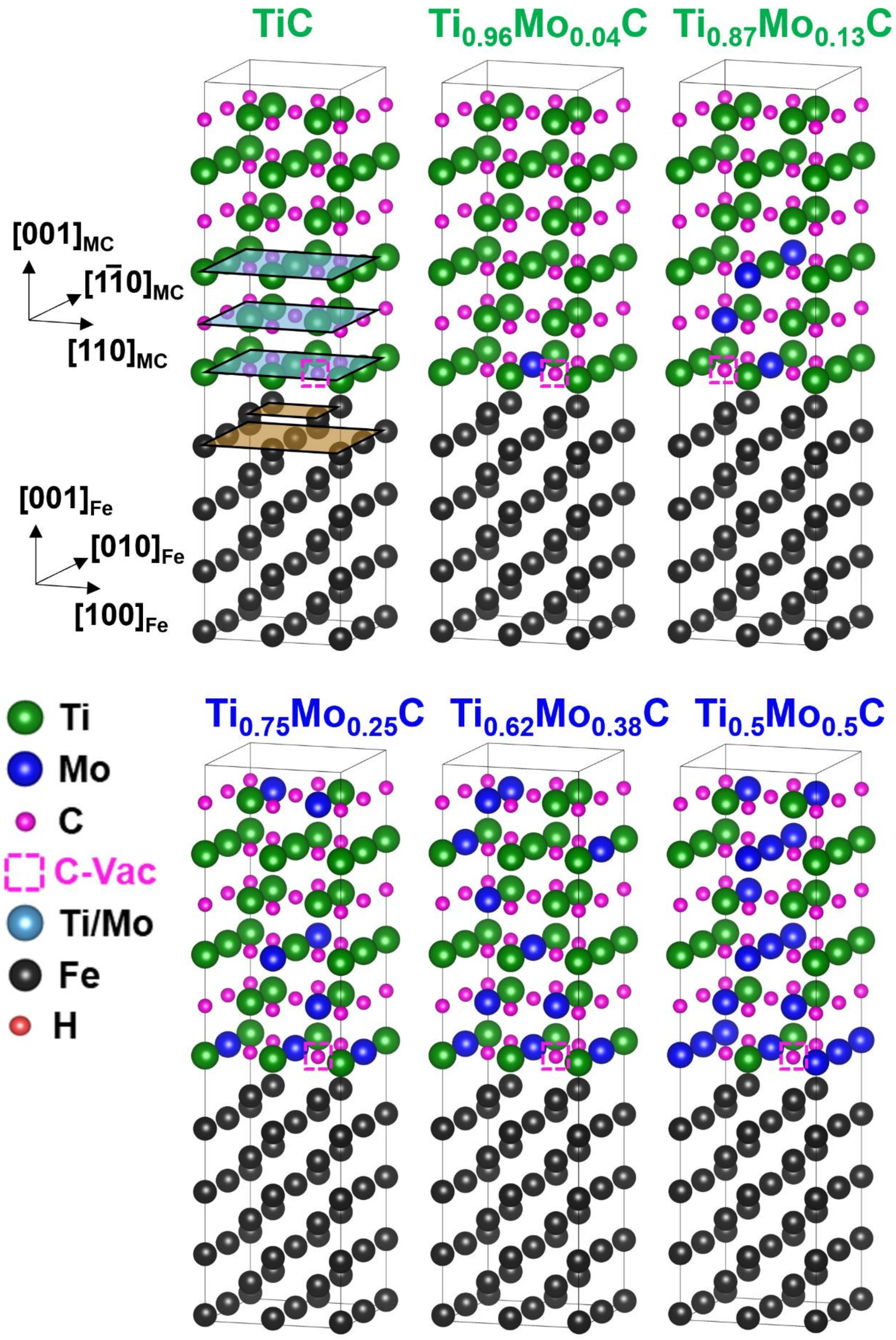

Supplementary Figure 1. Illustrations of the coherent interface models with different compositions of MC.

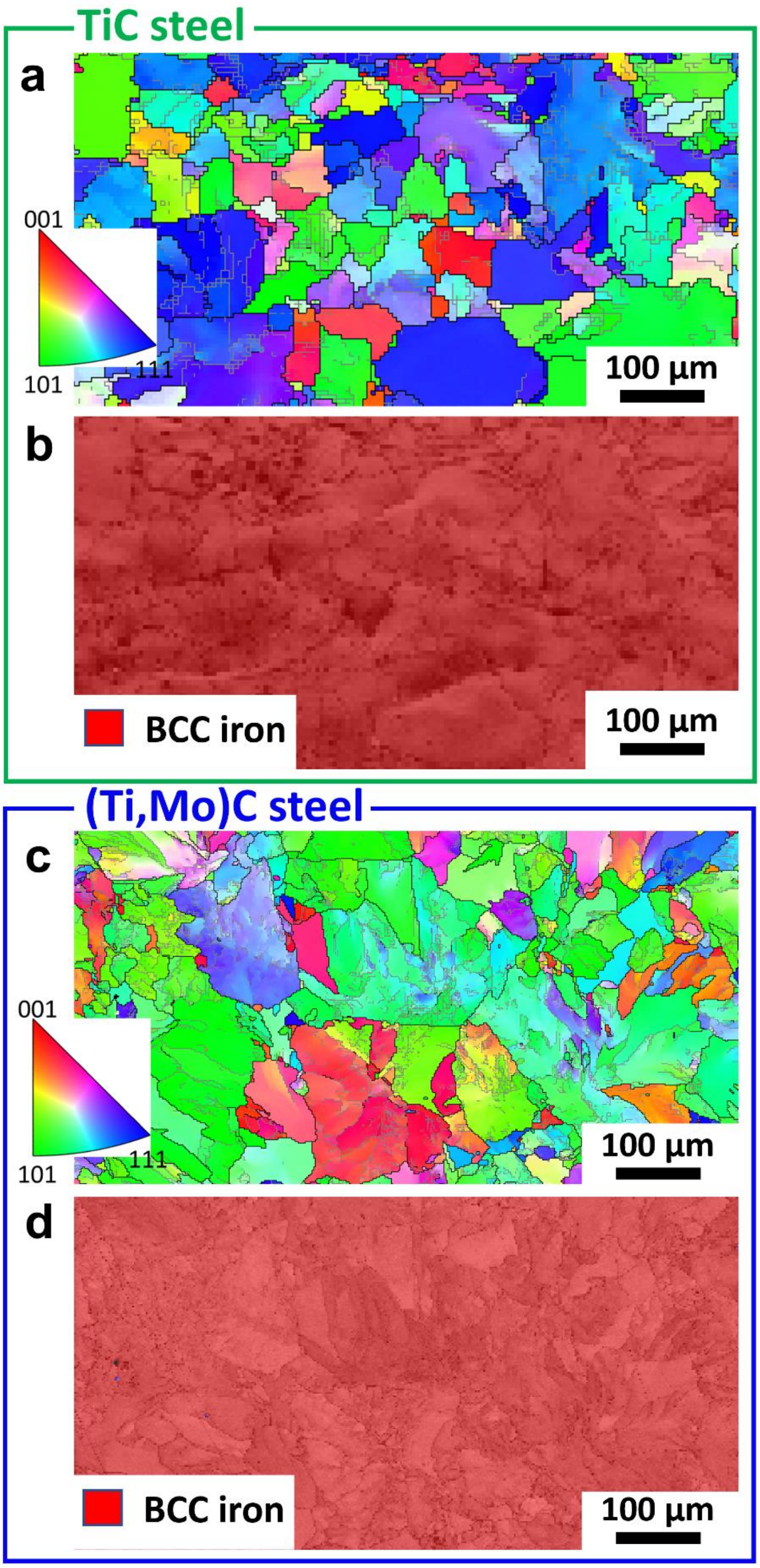

Supplementary Figure 2. Microstrucutre and phase composition of the steel samples. (a) and (b) are the SEM-EBSD map TiC steel (grain size: 195 μm) and (Ti,Mo)C steel (grain size: 140 μm), respectively. (c) and (d) are their corresponding phase maps, respectively.

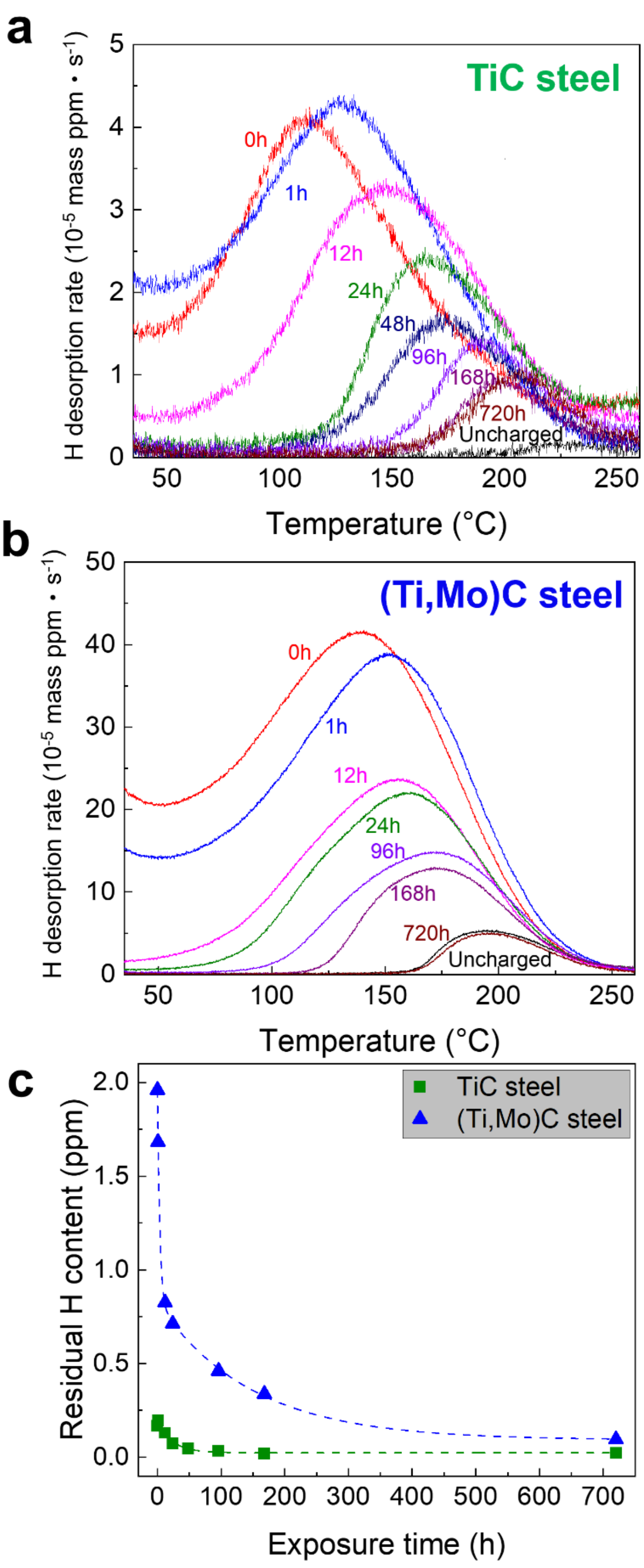

Supplementary Figure 3. TDS data for uncharged samples and hydrogen-charged samples with various desorption durations. (a) TiC steel. (b) (Ti,Mo)C steel. (c) The residual H content determined from desorption below 260 °C against the desorption duration after hydrogen charging

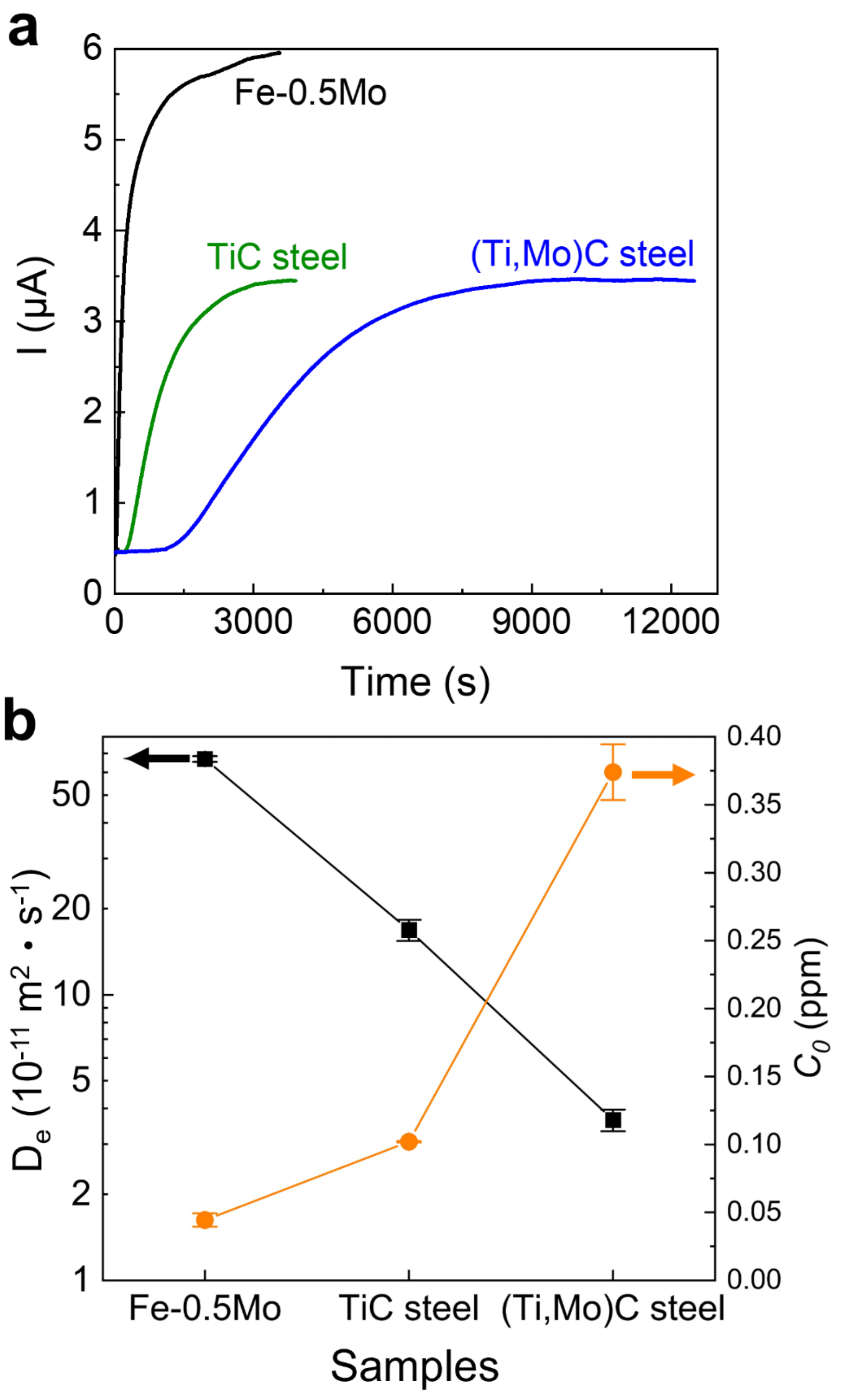

Supplementary Figure 4. Hydrogen permeation tests of the model interphase-precipitate steels in comparison with and the precipitate-free Fe-0.5Mo ferritic steel. (a) Permeation curves showing the current density I as a function of time and the retarded permeability of (Ti,Mo)C steel as compared to other samples. (b) The effective H diffusion coefficient, D_e, and the sub-surface hydrogen concentration, C_0, at the entry side of different samples. All error bars (standard deviations) were obtained by at least two measurements.

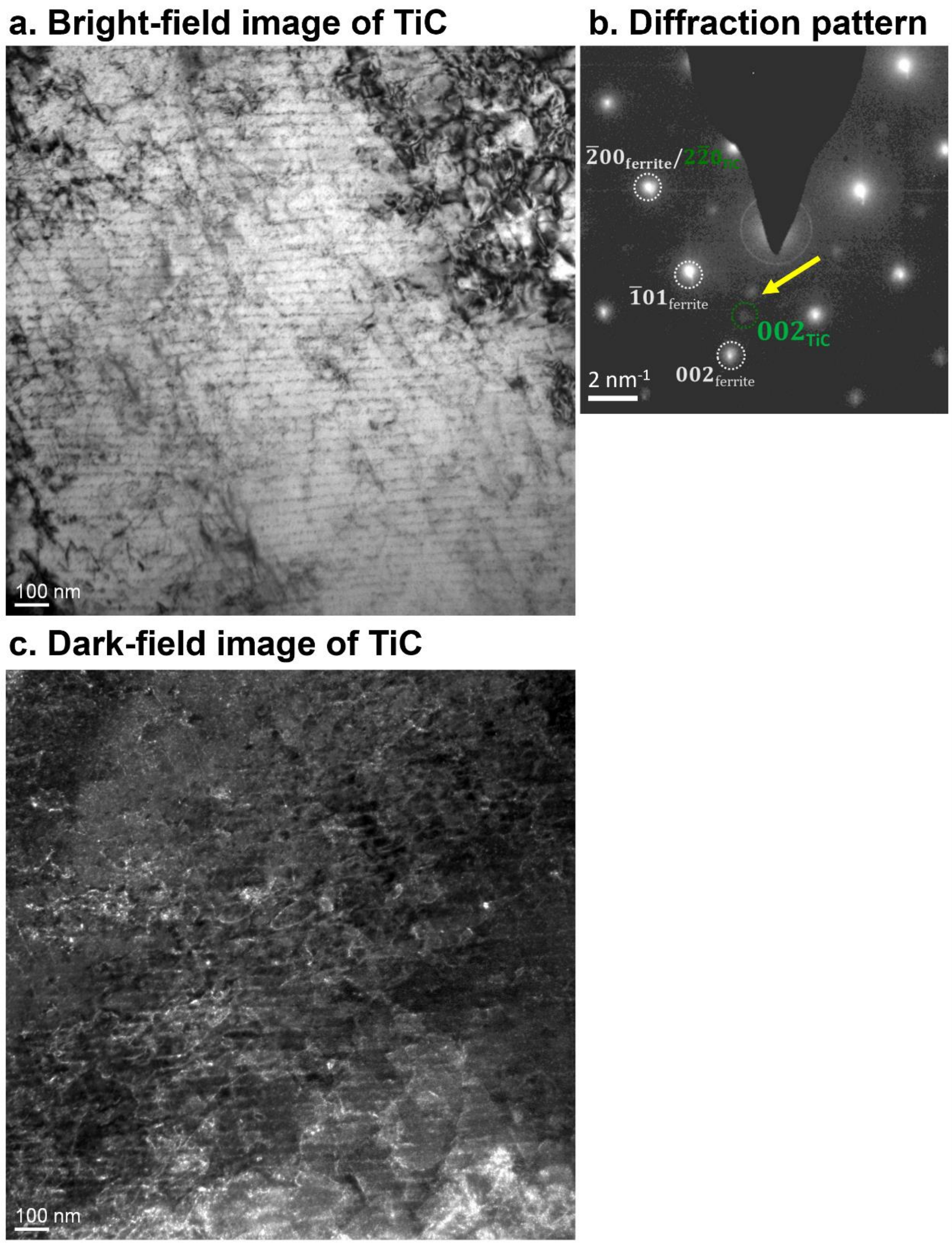

Supplementary Figure 5. TEM analysis of TiC. (a) Bright-field image. (b) Diffraction patterns from (a). (c) Dark-field image using the (002) diffraction spot of TiC as highlighted by the yellow arrow in (b).

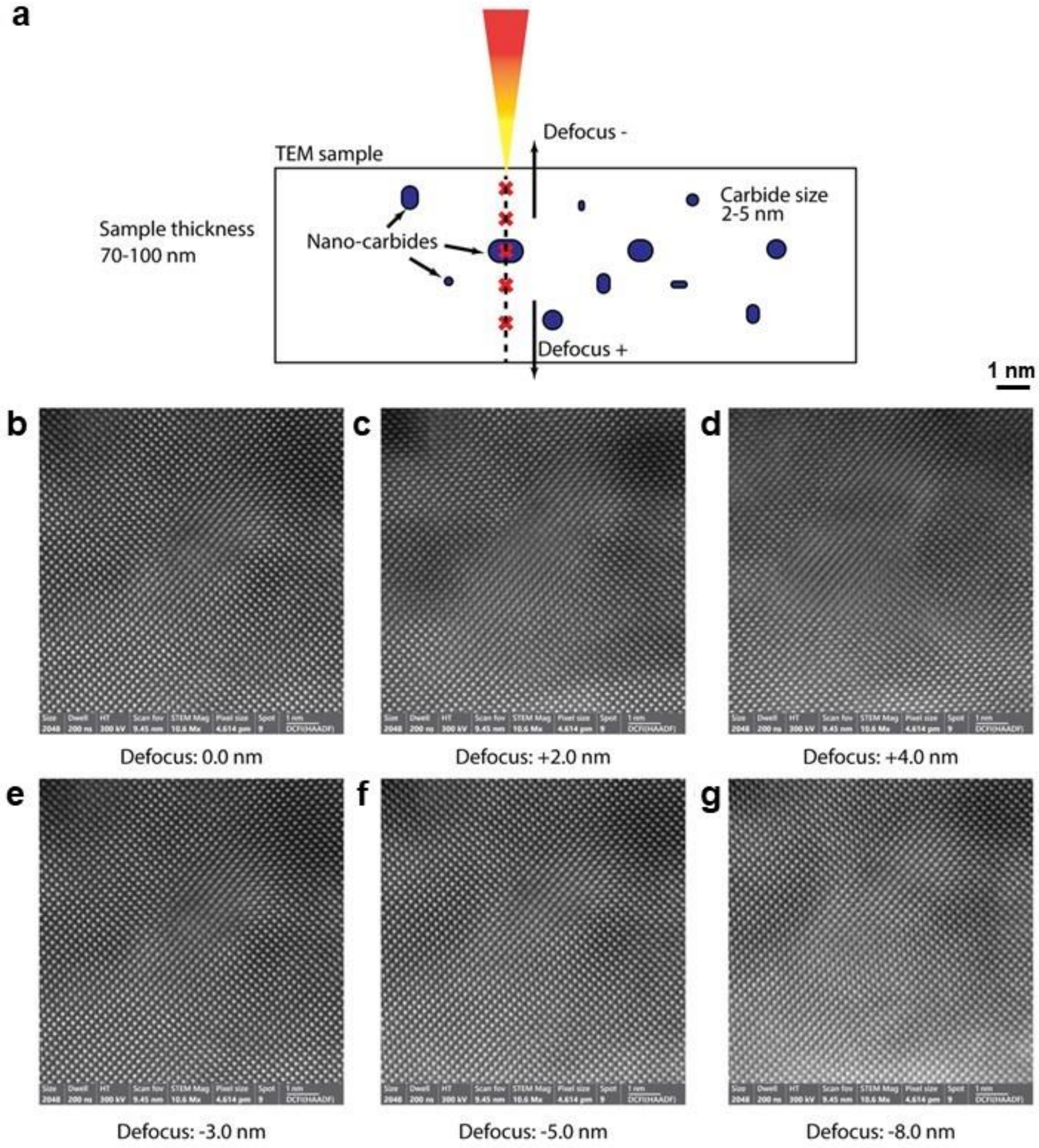

Supplementary Figure 6. Imaging condition for observing nanosized carbides using atomic-resolution STEM. (a) Schematic illustration showing the small MCs (2-5 nm) embedded in a STEM lift-out sample that is usually around 70-100 nm thick. The atomic structure of MCs can therefore overlap with the ferrite matrix. (b)-(g) shows the STEM images at various focal planes in the samples. (b) is in-focus, where the carbide atom column can be seen. Whereas (c) and (d) are over-focused and (e), (f), and (g) are under focused, where the atomic structures relating to the carbides are not visible.

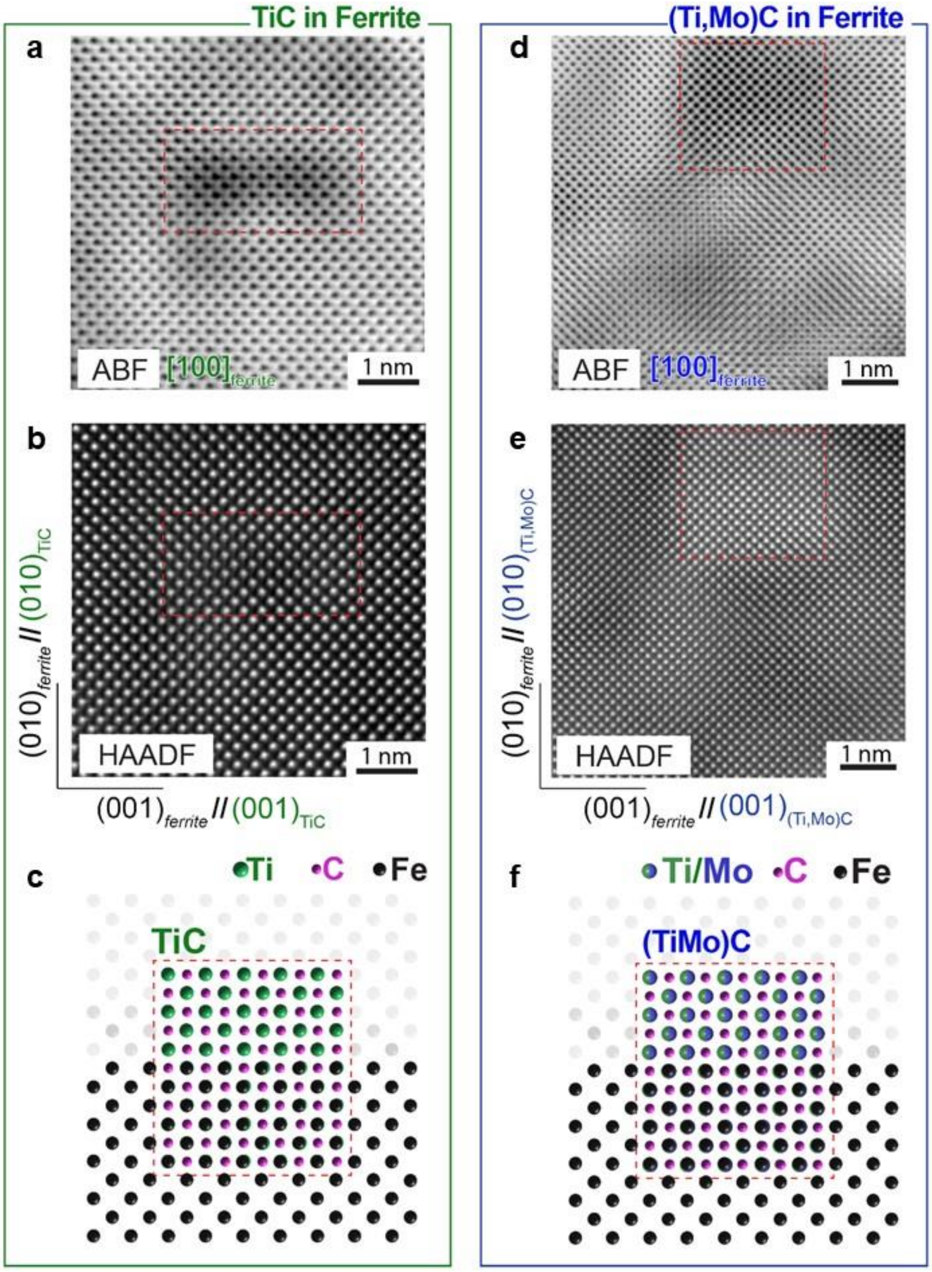

Supplementary Figure 7. Additional atomic-resolution STEM images showing the carbides along [100]$_{ferrite}$ zone axis, corresponding to Fig. 3. (a)-(c) are from the TiC steel, and (d)-(f) are from the (Ti,Mo)C steel. (a) and (d) are ABF-STEM images, showing the atomic columns in the carbides fully coincide with the ferrite lattice along this zone axis. (b) and (e) are the corresponding high-angle annular dark-field (HAADF) STEM images. The red squares highlight that the light Ti atoms in the TiC are slightly darker than the Fe atoms in the ferrite matrix, whereas the heavy Mo atoms are brighter than the Fe atoms, due to their difference in atomic mass. (c) and (f) are corresponding atomic models illustrating the atomic structure under observation, showing the simulated structure of the carbides (colored spheres) embedded in the BCC ferrite matrix (black spheres).

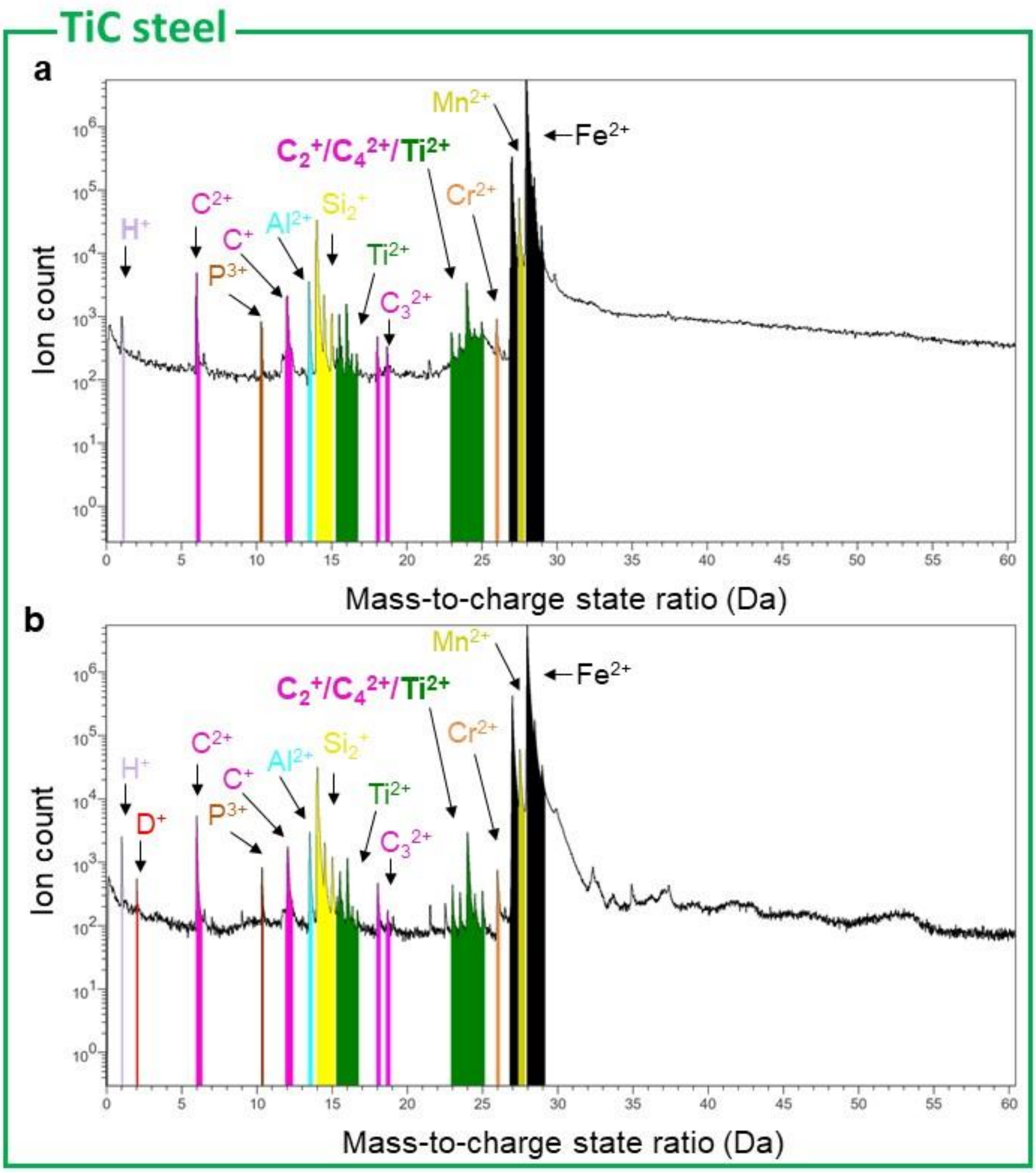

Supplementary Figure 8. APT mass spectra from the (a) uncharged and (b) deuterium-charged TiC steel samples, corresponding to Fig. 4.

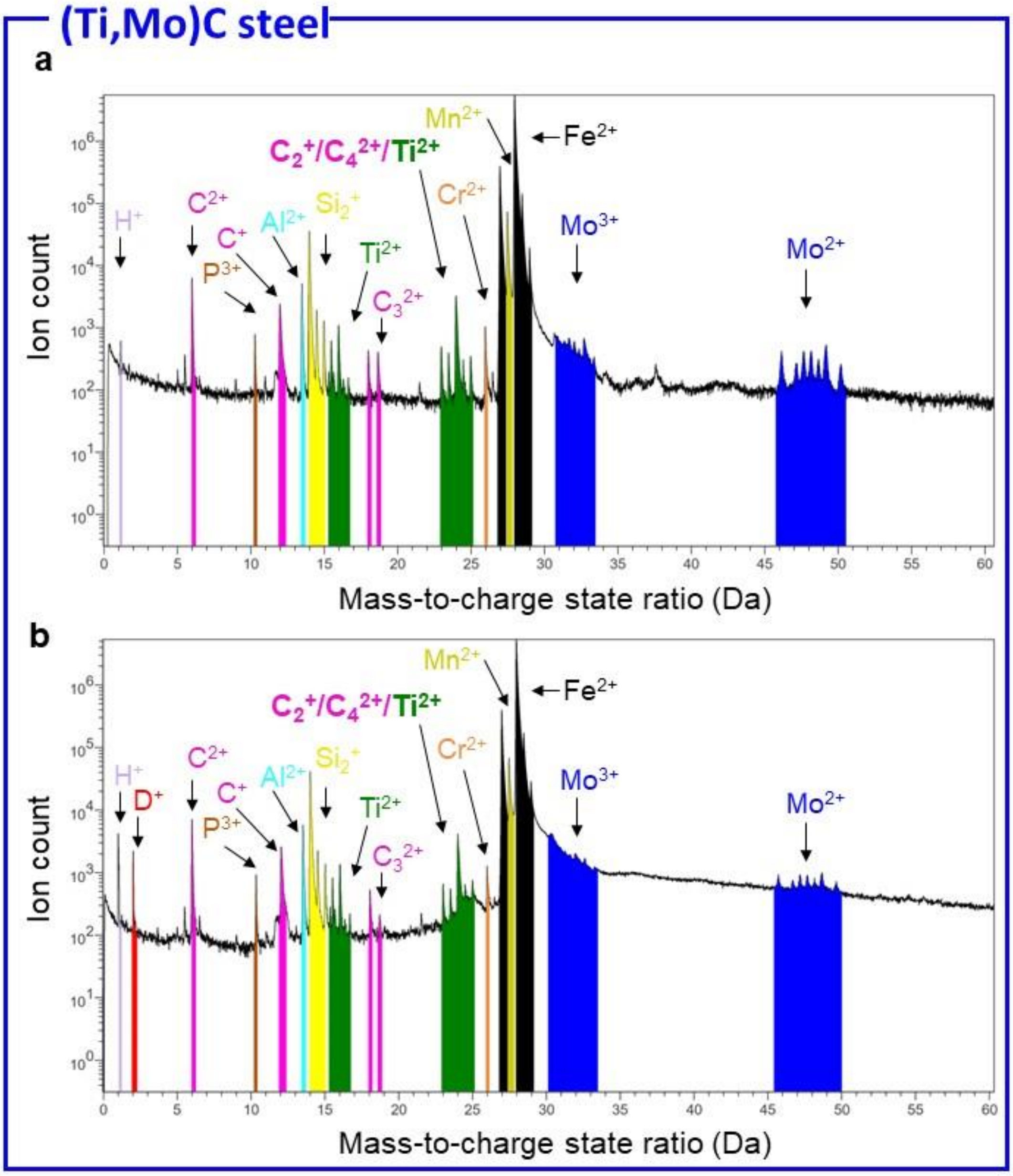

Supplementary Figure 9. APT mass spectra from the (a) uncharged and (b) deuterium-charged (Ti,Mo)C steel samples, corresponding to Fig. 5.

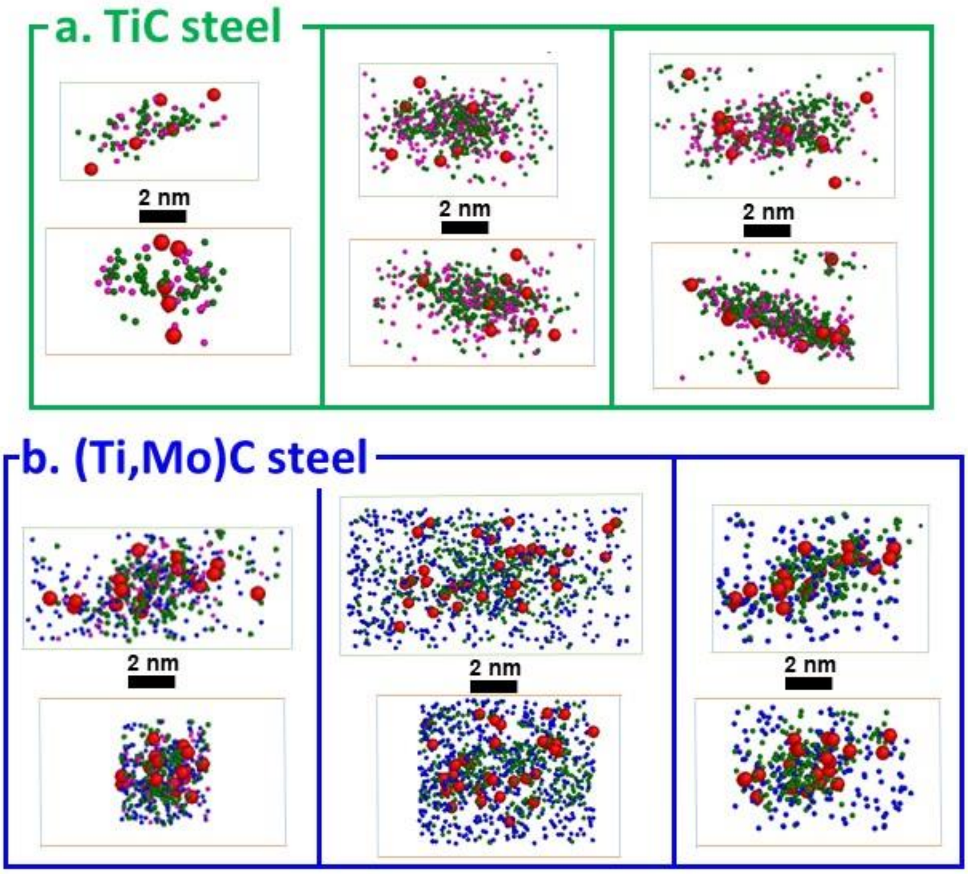

Supplementary Figure 10. Additional atomic views of (a) TiCs and (b) (Ti,Mo)Cs with trapped D atoms (red spheres), corresponding to Figs. 4 and 5, respectively.

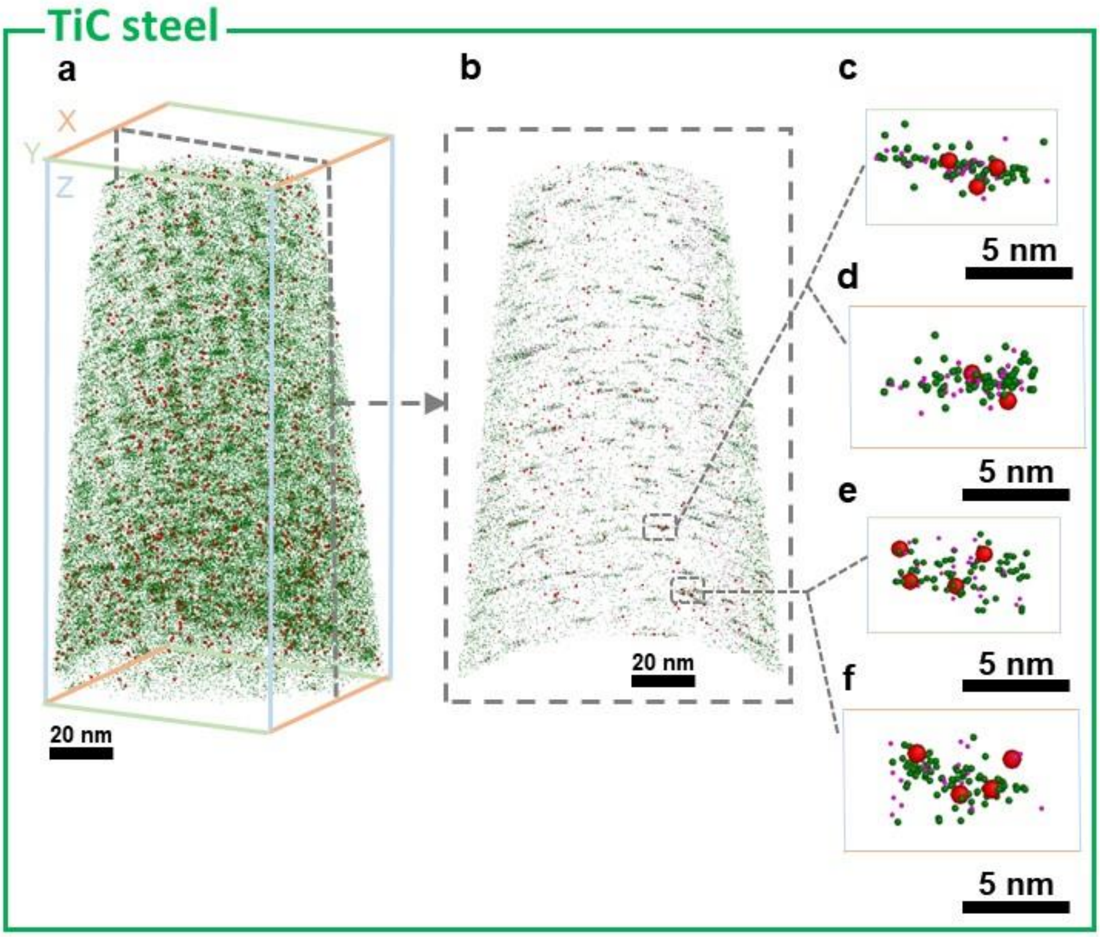

Supplementary Figure 11. Additional APT analysis of D-charged TiC steel using the same condition as the results in Fig. 4. (a) Oerall reconstructed data displaying only Ti (green), C (magenta), and D (red). (b) 2-D slice from the area highlighted by the grey square in panel (a). (c)-(f) Magnified views of two carbides selected from panel (b), from two different perspectives.

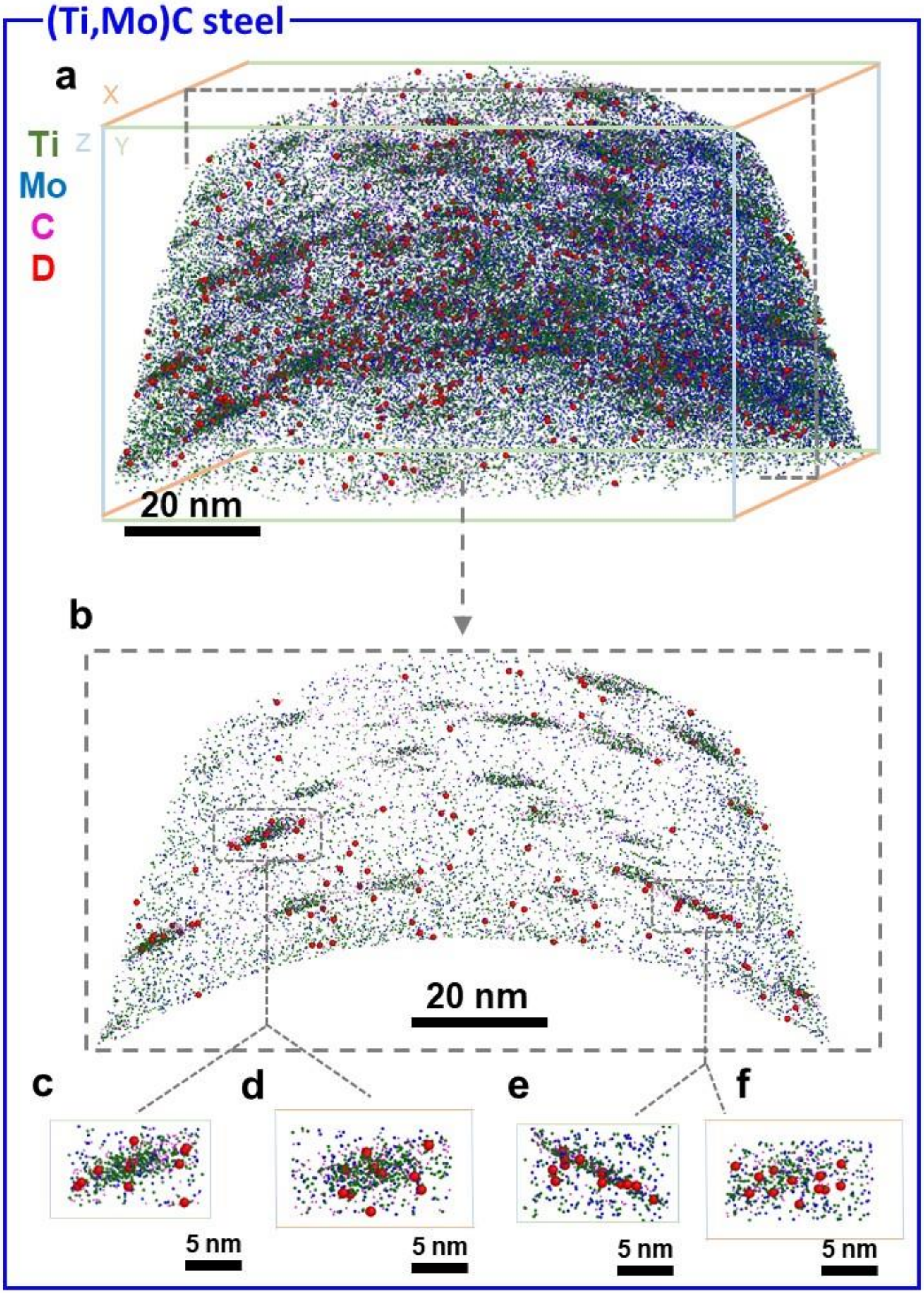

Supplementary Figure 12. Additional APT analysis of D-charged (Ti,Mo)C steel using the same condition as the results in Fig. 5. (a) Oerall reconstructed data displaying only Ti (green), Mo (blue), C (magenta), and D (red). (b) 2-D slice from the area highlighted by the grey square in panel (a). (c)-(f) Magnified views of two carbides selected from panel (b), from two different perspectives.

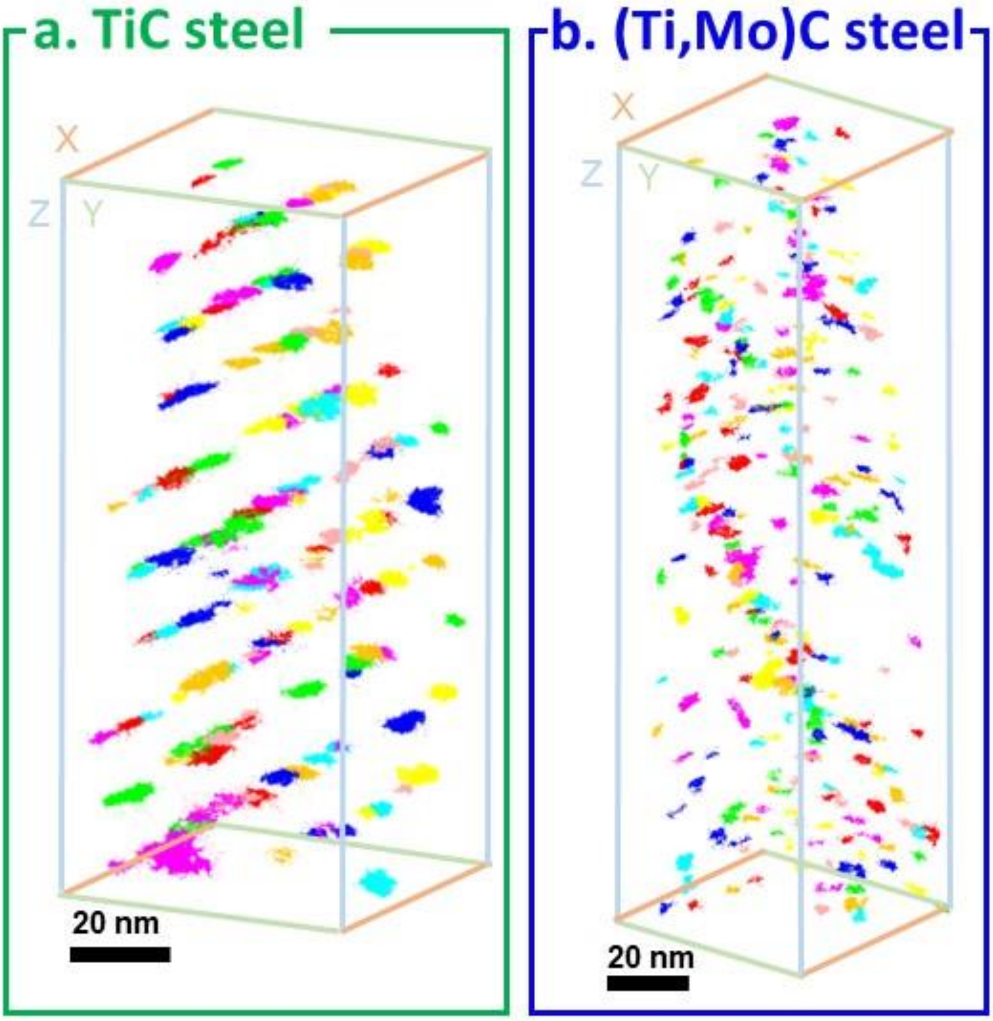

Supplementary Figure 13. Visualizations of the defined clusters in (a) TiC steel and (b) (Ti,Mo)C steel using the Cluster Analysis tool in the APT software.
