# Supplementary material for: Engineering metal-carbide hydrogen traps in steels
Source: Nat Commun. 2024 Jan 25;15:724. doi: 10.1038/s41467-024-45017-4 (PMC10808193; doi:10.1038/s41467-024-45017-4)
Supplement: Supplementary file 3 — Description of additional supplementary files [file 41467_2024_45017_MOESM3_ESM.docx]

### Supplementary Movie 1. Animated APT data of deuterated TiC steel corresponding to Fig. 4. A 10-nm slice flyover view of the 3-D atom map with deuterium in red, titanium in green, and carbon in magenta.

### Supplementary Movie 2. Animated APT data of deuterated (Ti,Mo)C steel corresponding to Fig. 5. b 10-nm slice flyover view of the 3-D atom map with deuterium in red, titanium in green, molybdenum in blue, and carbon in magenta.
